# 1-[4-(4-Isopropyl­phen­yl)-6-methyl-2-sulfanyl­idene-1,2,3,4-tetra­hydro­pyrimidin-5-yl]ethanone

**DOI:** 10.1107/S1600536812034046

**Published:** 2012-08-04

**Authors:** N. Anuradha, A. Thiruvalluvar, S. Chitra, D. Devanathan, Oluwaseun O. Falola, R.J. Butcher

**Affiliations:** a PG Research Department of Physics, Rajah Serfoji Government College (Autonomous), Thanjavur 613 005, Tamilnadu, India; b Department of Chemistry, K.S.R. College of Engineering, K.S.R. Kalvi Nagar, Tiruchengode 637 215, Tamilnadu, India; c Department of Chemistry, Government Arts College, C. Mutlur 608 102, Chidambaram, Tamilnadu, India; d Department of Chemistry, Howard University, 525 College Street NW, Washington, DC 20059, USA

## Abstract

In the title mol­ecule, C_16_H_20_N_2_OS, the heterocyclic ring adopts a slightly distorted flattened boat conformation, and the plane through the four coplanar atoms makes a dihedral angle of 86.98 (6)° with the benzene ring. The thione, acetyl and methyl groups lie on the opposite side of the heterocyclic mean plane to the isopropylphenyl group which has an axial orientation. A weak intra­molecular C—H⋯O hydrogen bond is observed. In the crystal, molecules are linked *via* N—H⋯O, N—H⋯S and C—H⋯S hydrogen bonds.

## Related literature

For chemical and biological applications and for the closely related crystal structures of the chloro and fluoro derivatives, see: Anuradha *et al.* (2009 ▶, 2012 ▶).
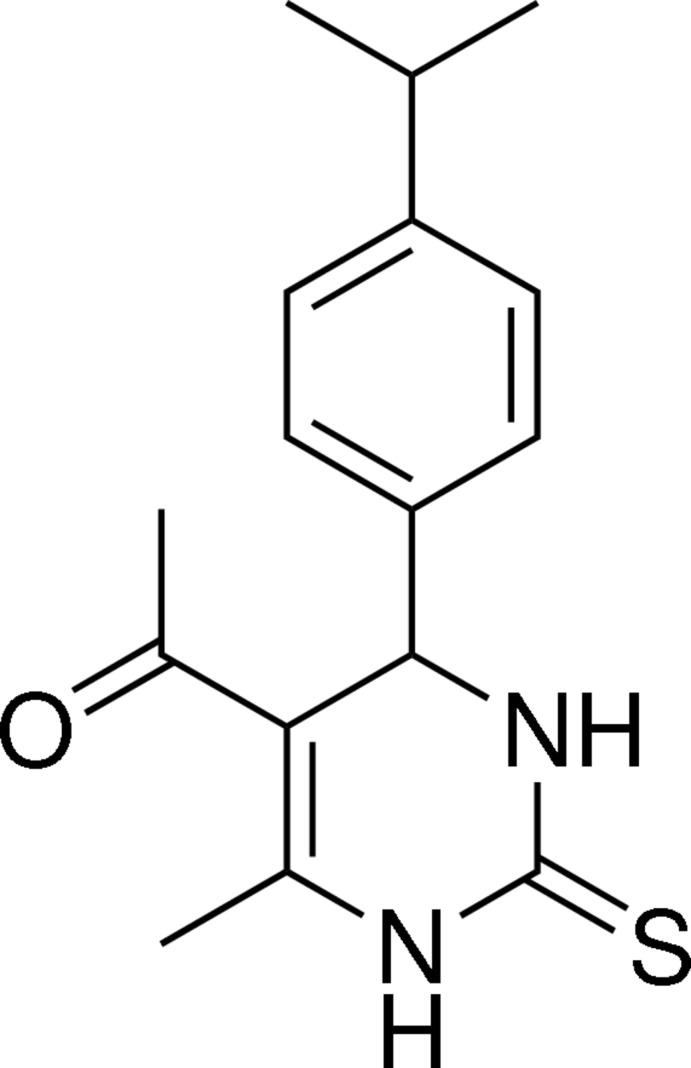


## Experimental

### 

#### Crystal data

C_16_H_20_N_2_OS
*M* *_r_* = 288.41
Monoclinic, 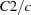

*a* = 26.8413 (5) Å
*b* = 9.5657 (2) Å
*c* = 12.0764 (2) Å
β = 90.370 (2)°
*V* = 3100.62 (10) Å^3^
*Z* = 8
Cu *K*α radiation
μ = 1.83 mm^−1^
*T* = 123 K
0.49 × 0.23 × 0.18 mm

#### Data collection

Oxford Diffraction Xcalibur Ruby Gemini diffractometer
Absorption correction: multi-scan (*CrysAlis PRO*; Agilent, 2012 ▶) *T*_min_ = 0.831, *T*_max_ = 1.000
5607 measured reflections
3042 independent reflections
2776 reflections with *I* > 2σ(*I*)
*R*_int_ = 0.024

#### Refinement

*R*[*F* ^2^ > 2σ(*F* ^2^)] = 0.041
*wR*(*F* ^2^) = 0.113
*S* = 1.05
3042 reflections
193 parameters
H atoms treated by a mixture of independent and constrained refinement
Δρ_max_ = 0.42 e Å^−3^
Δρ_min_ = −0.39 e Å^−3^

### 

Data collection: *CrysAlis PRO* (Agilent, 2012 ▶); cell refinement: *CrysAlis PRO*; data reduction: *CrysAlis PRO*; program(s) used to solve structure: *SHELXS97* (Sheldrick, 2008 ▶); program(s) used to refine structure: *SHELXL97* (Sheldrick, 2008 ▶); molecular graphics: *ORTEP-3* (Farrugia, 1997 ▶) and *PLATON* (Spek, 2009 ▶); software used to prepare material for publication: *PLATON*.

## Supplementary Material

Crystal structure: contains datablock(s) global, I. DOI: 10.1107/S1600536812034046/wn2487sup1.cif

Structure factors: contains datablock(s) I. DOI: 10.1107/S1600536812034046/wn2487Isup2.hkl

Supplementary material file. DOI: 10.1107/S1600536812034046/wn2487Isup3.cdx

Supplementary material file. DOI: 10.1107/S1600536812034046/wn2487Isup4.cml

Additional supplementary materials:  crystallographic information; 3D view; checkCIF report

## Figures and Tables

**Table 1:** Hydrogen-bond geometry (Å, °)

*D*—H⋯*A*	*D*—H	H⋯*A*	*D*⋯*A*	*D*—H⋯*A*
N1—H1⋯O51^i^	0.83 (2)	2.12 (2)	2.9445 (17)	174 (2)
N3—H3⋯S2^ii^	0.840 (19)	2.56 (2)	3.3481 (13)	156.9 (18)
C61—H61*A*⋯O51	0.98	2.22	2.9148 (19)	127
C61—H61*B*⋯S2^iii^	0.98	2.86	3.7145 (16)	146

Symmetry codes: (i) 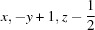; (ii) 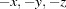; (iii) 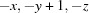.

**Fig. 1.**
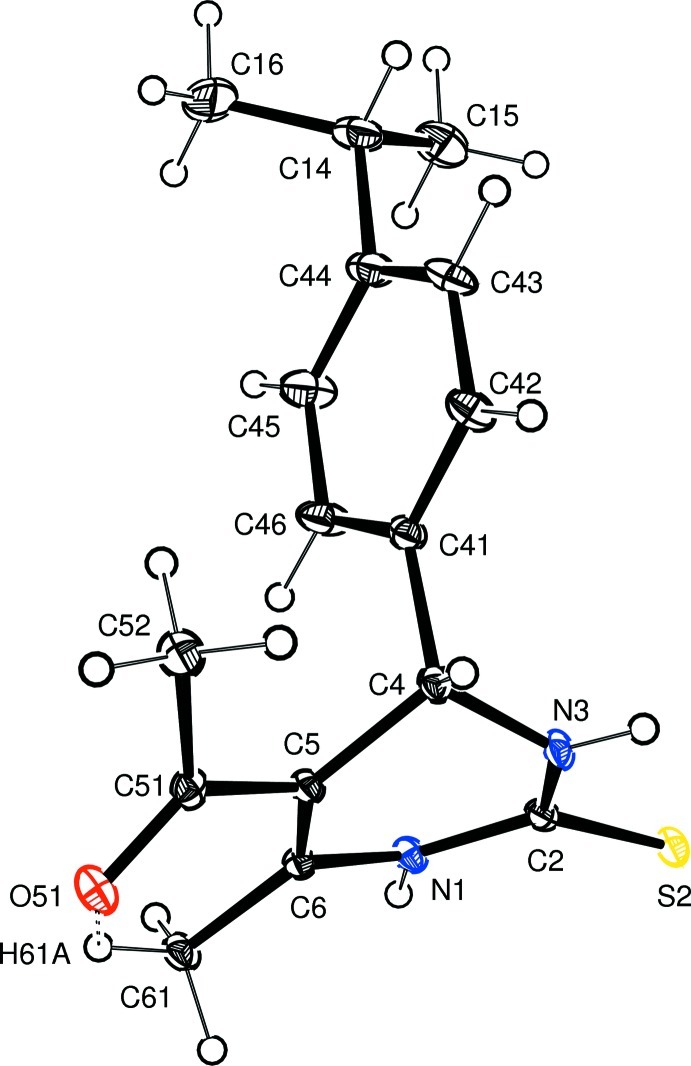
The molecular structure of the title compound, with displacement ellipsoids drawn at the 30% probability level. H atoms are shown as small spheres of arbitrary radius. Dashed lines indicate the intramolecular C—H···O hydrogen bond.

**Fig. 2.**
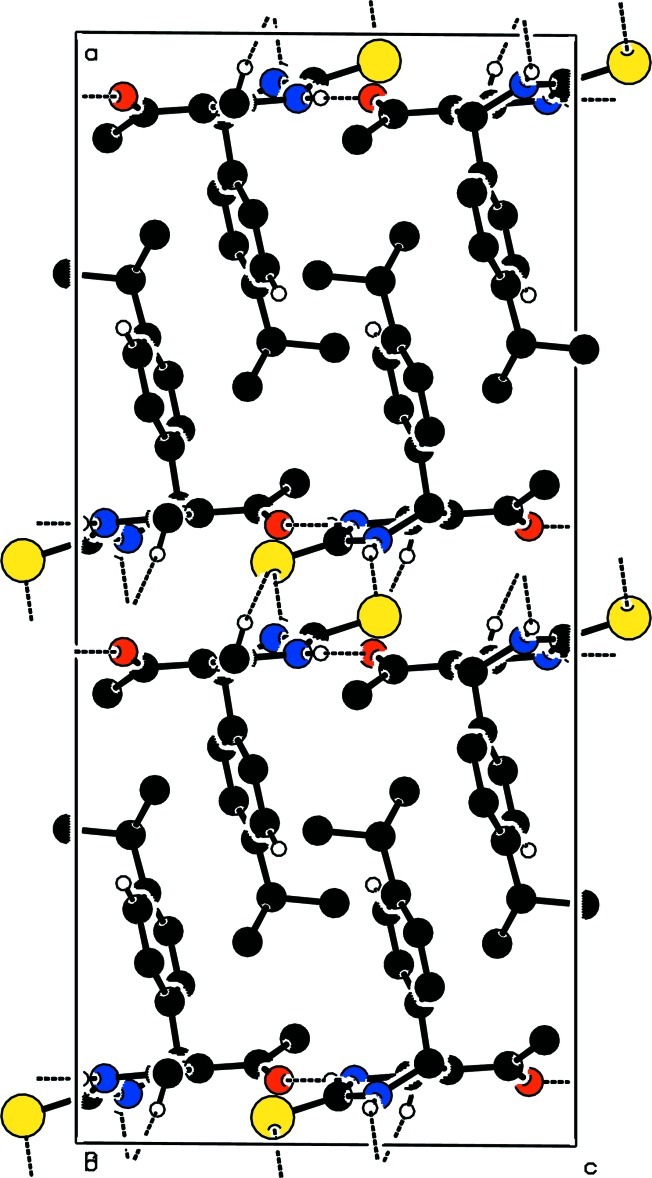
The packing of the title compound, viewed down the b axis. Dashed lines indicate hydrogen bonds. H atoms not involved in hydrogen bonding have been omitted.

## supplementary crystallographic information

### Comment

As part of our investigations of dihydropyrimidine derivatives (Anuradha *et al.*, 2009, 2012) to compare their chemical and biological activities, we have undertaken the X-ray crystal structure analysis of the title compound.

In the title molecule, C_16_H_20_N_2_OS (Fig.1), the heterocyclic ring adopts a slightly distorted flattened boat conformation, and the plane through the four coplanar atoms( C2, N3, C5 and C6) makes a dihedral angle of 86.98 (6)° with the benzene ring. The thione, acetyl and methyl groups have equatorial orientations with respect to the attached heterocyclic ring, whereas the isopropylphenyl group has an axial orientation.

Intermolecular N1—H1···O51, N3—H3···S2 and C61—H61B···S2 hydrogen bonds are found in the crystal structure. A weak intramolecular C61—H61A···O51 hydrogen bond is also observed (Fig. 2, Table 1).

### Experimental

A solution of acetylacetone (1.0012 g, 0.01 mol), 4-isopropylbenzaldehyde (1.48 g, 0.01 mol) and thiourea (1.14 g, 0.015 mol) was heated under reflux in the presence of calcium fluoride (0.07 g, 0.001 mol) for 2 h (monitored by TLC). After completion of the reaction, the reaction mixture was cooled to room temperature and poured into crushed ice. The solid product was filtered under suction and purified by recrystallization from hot methanol to give the product in pure form. Yield 1.86 g (93%).

### Refinement

The two N-bound H atoms were located in a difference Fourier map and refined freely; N1—H1 = 0.83 (2) Å and N3—H3 = 0.840 (19) Å. The remaining H atoms were positioned geometrically and allowed to ride on their parent atoms, with C*sp*^2^—H = 0.95, *C*(methyl)—H = 0.98, and C(methine)—H = 1.00 Å; *U*_iso_(H) = k*U*_eq_(C), where k = 1.5 for methyl H and 1.2 for all other H atoms.

### Crystal data

**Table:**

C_16_H_20_N_2_OS	*F*(000) = 1232
*M**_r_* = 288.41	*D*_x_ = 1.236 Mg m^−^^3^
Monoclinic, *C*2/*c*	Melting point: 486 K
Hall symbol: -C 2yc	Cu *K*α radiation, λ = 1.54184 Å
*a* = 26.8413 (5) Å	Cell parameters from 3841 reflections
*b* = 9.5657 (2) Å	θ = 3.3–73.5°
*c* = 12.0764 (2) Å	µ = 1.83 mm^−^^1^
β = 90.370 (2)°	*T* = 123 K
*V* = 3100.62 (10) Å^3^	Prism, colourless
*Z* = 8	0.49 × 0.23 × 0.18 mm

### Data collection

**Table:**

Oxford Diffraction Xcalibur Ruby Gemini diffractometer	3042 independent reflections
Radiation source: Enhance (Cu) X-ray Source	2776 reflections with *I* > 2σ(*I*)
Graphite monochromator	*R*_int_ = 0.024
Detector resolution: 10.5081 pixels mm^-1^	θ_max_ = 73.6°, θ_min_ = 3.3°
ω scans	*h* = −30→33
Absorption correction: multi-scan (*CrysAlis PRO*; Agilent, 2012)	*k* = −11→10
*T*_min_ = 0.831, *T*_max_ = 1.000	*l* = −10→14
5607 measured reflections	

### Refinement

**Table:**

Refinement on *F*^2^	Primary atom site location: structure-invariant direct methods
Least-squares matrix: full	Secondary atom site location: difference Fourier map
*R*[*F*^2^ > 2σ(*F*^2^)] = 0.041	Hydrogen site location: inferred from neighbouring sites
*wR*(*F*^2^) = 0.113	H atoms treated by a mixture of independent and constrained refinement
*S* = 1.05	*w* = 1/[σ^2^(*F*_o_^2^) + (0.0684*P*)^2^ + 1.9078*P*] where *P* = (*F*_o_^2^ + 2*F*_c_^2^)/3
3042 reflections	(Δ/σ)_max_ = 0.001
193 parameters	Δρ_max_ = 0.42 e Å^−^^3^
0 restraints	Δρ_min_ = −0.39 e Å^−^^3^

### Special details

**Table:**

Geometry. Bond distances, angles *etc*. have been calculated using the rounded fractional coordinates. All su's are estimated from the variances of the (full) variance-covariance matrix. The cell e.s.d.'s are taken into account in the estimation of distances, angles and torsion angles
Refinement. Refinement of *F*^2^ against ALL reflections. The weighted *R*-factor *wR* and goodness of fit *S* are based on *F*^2^, conventional *R*-factors *R* are based on *F*, with *F* set to zero for negative *F*^2^. The threshold expression of *F*^2^ > 2σ(*F*^2^) is used only for calculating *R*-factors(gt) *etc*. and is not relevant to the choice of reflections for refinement. *R*-factors based on *F*^2^ are statistically about twice as large as those based on *F*, and *R*- factors based on ALL data will be even larger.

### Fractional atomic coordinates and isotropic or equivalent isotropic displacement parameters (Å^2^)

**Table:**

	*x*	*y*	*z*	*U*_iso_*/*U*_eq_	
S2	0.02408 (1)	0.17969 (4)	−0.10663 (3)	0.0195 (1)	
O51	0.05759 (4)	0.41789 (12)	0.40606 (9)	0.0236 (3)	
N1	0.05834 (5)	0.34011 (14)	0.05675 (10)	0.0163 (3)	
N3	0.04445 (5)	0.11271 (13)	0.10324 (10)	0.0174 (3)	
C2	0.04342 (5)	0.20984 (16)	0.02461 (12)	0.0157 (4)	
C4	0.07362 (5)	0.12832 (15)	0.20639 (12)	0.0163 (4)	
C5	0.06781 (5)	0.27819 (15)	0.24566 (12)	0.0148 (4)	
C6	0.06273 (5)	0.37919 (15)	0.16783 (12)	0.0154 (4)	
C14	0.27761 (7)	−0.0514 (2)	0.10895 (16)	0.0335 (6)	
C15	0.28259 (8)	−0.0600 (2)	−0.01678 (18)	0.0417 (7)	
C16	0.31816 (8)	0.0428 (3)	0.1572 (2)	0.0553 (8)	
C41	0.12759 (6)	0.08478 (16)	0.18712 (12)	0.0184 (4)	
C42	0.14242 (7)	−0.05178 (17)	0.20769 (14)	0.0255 (5)	
C43	0.19116 (7)	−0.09414 (18)	0.18449 (15)	0.0294 (5)	
C44	0.22550 (6)	−0.00268 (19)	0.14005 (14)	0.0268 (5)	
C45	0.21077 (7)	0.1352 (2)	0.12272 (17)	0.0336 (6)	
C46	0.16255 (7)	0.17846 (18)	0.14600 (16)	0.0295 (5)	
C51	0.07174 (5)	0.30757 (16)	0.36521 (12)	0.0172 (4)	
C52	0.09518 (7)	0.19628 (17)	0.43753 (13)	0.0238 (4)	
C61	0.06312 (6)	0.53426 (16)	0.18341 (13)	0.0205 (4)	
H1	0.0578 (8)	0.404 (2)	0.0103 (18)	0.027 (5)*	
H3	0.0331 (8)	0.033 (2)	0.0883 (16)	0.022 (5)*	
H4	0.05908	0.06499	0.26360	0.0195*	
H14	0.28252	−0.14749	0.13978	0.0401*	
H15A	0.25674	−0.12199	−0.04673	0.0625*	
H15B	0.31551	−0.09716	−0.03547	0.0625*	
H15C	0.27872	0.03349	−0.04881	0.0625*	
H16A	0.31439	0.13744	0.12736	0.0829*	
H16B	0.35096	0.00585	0.13732	0.0829*	
H16C	0.31517	0.04545	0.23804	0.0829*	
H42	0.11939	−0.11679	0.23764	0.0305*	
H43	0.20084	−0.18777	0.19963	0.0353*	
H45	0.23407	0.20068	0.09452	0.0403*	
H46	0.15335	0.27312	0.13370	0.0353*	
H52A	0.09405	0.22594	0.51515	0.0357*	
H52B	0.12992	0.18230	0.41561	0.0357*	
H52C	0.07678	0.10845	0.42872	0.0357*	
H61A	0.07008	0.55610	0.26127	0.0308*	
H61B	0.03056	0.57263	0.16234	0.0308*	
H61C	0.08898	0.57568	0.13681	0.0308*	

### Atomic displacement parameters (Å^2^)

**Table:**

	*U*^11^	*U*^22^	*U*^33^	*U*^12^	*U*^13^	*U*^23^
S2	0.0271 (2)	0.0143 (2)	0.0171 (2)	0.0000 (1)	−0.0027 (1)	−0.0011 (1)
O51	0.0312 (6)	0.0183 (6)	0.0212 (5)	0.0007 (5)	0.0029 (4)	−0.0047 (4)
N1	0.0205 (6)	0.0117 (6)	0.0168 (6)	0.0001 (5)	0.0005 (5)	0.0016 (5)
N3	0.0226 (6)	0.0101 (6)	0.0194 (6)	−0.0027 (5)	−0.0022 (5)	−0.0006 (5)
C2	0.0159 (7)	0.0132 (7)	0.0181 (7)	0.0017 (5)	0.0011 (5)	−0.0003 (5)
C4	0.0190 (7)	0.0129 (7)	0.0169 (7)	−0.0008 (5)	−0.0005 (5)	0.0009 (5)
C5	0.0143 (6)	0.0123 (7)	0.0179 (7)	0.0000 (5)	0.0005 (5)	−0.0011 (5)
C6	0.0123 (6)	0.0145 (7)	0.0195 (7)	0.0000 (5)	0.0009 (5)	−0.0023 (5)
C14	0.0285 (9)	0.0315 (10)	0.0404 (10)	0.0150 (8)	0.0036 (7)	−0.0022 (8)
C15	0.0407 (11)	0.0405 (12)	0.0440 (11)	0.0109 (9)	0.0115 (9)	−0.0081 (9)
C16	0.0253 (10)	0.0666 (16)	0.0738 (16)	0.0188 (10)	−0.0064 (10)	−0.0274 (13)
C41	0.0212 (7)	0.0157 (7)	0.0183 (7)	0.0032 (6)	−0.0008 (5)	−0.0001 (5)
C42	0.0310 (8)	0.0164 (8)	0.0291 (8)	0.0032 (7)	0.0035 (6)	0.0025 (6)
C43	0.0355 (9)	0.0177 (8)	0.0351 (9)	0.0121 (7)	0.0026 (7)	0.0021 (7)
C44	0.0258 (8)	0.0260 (9)	0.0286 (8)	0.0099 (7)	0.0008 (6)	−0.0015 (7)
C45	0.0250 (8)	0.0262 (10)	0.0497 (11)	0.0050 (7)	0.0091 (7)	0.0095 (8)
C46	0.0240 (8)	0.0187 (9)	0.0458 (11)	0.0059 (7)	0.0065 (7)	0.0080 (7)
C51	0.0154 (7)	0.0163 (7)	0.0198 (7)	−0.0041 (5)	0.0014 (5)	0.0006 (5)
C52	0.0312 (8)	0.0216 (8)	0.0186 (7)	0.0011 (7)	−0.0026 (6)	0.0019 (6)
C61	0.0268 (8)	0.0130 (7)	0.0218 (7)	0.0025 (6)	−0.0021 (6)	0.0001 (6)

### Geometric parameters (Å, º)

**Table:**

S2—C2	1.6894 (15)	C45—C46	1.389 (3)
O51—C51	1.2262 (19)	C51—C52	1.512 (2)
N1—C2	1.365 (2)	C4—H4	1.0000
N1—C6	1.3968 (19)	C14—H14	1.0000
N3—C2	1.3287 (19)	C15—H15A	0.9800
N3—C4	1.4746 (19)	C15—H15B	0.9800
N1—H1	0.83 (2)	C15—H15C	0.9800
N3—H3	0.840 (19)	C16—H16A	0.9800
C4—C41	1.527 (2)	C16—H16B	0.9800
C4—C5	1.518 (2)	C16—H16C	0.9800
C5—C51	1.474 (2)	C42—H42	0.9500
C5—C6	1.354 (2)	C43—H43	0.9500
C6—C61	1.495 (2)	C45—H45	0.9500
C14—C44	1.524 (2)	C46—H46	0.9500
C14—C15	1.527 (3)	C52—H52A	0.9800
C14—C16	1.526 (3)	C52—H52B	0.9800
C41—C46	1.392 (2)	C52—H52C	0.9800
C41—C42	1.388 (2)	C61—H61A	0.9800
C42—C43	1.400 (3)	C61—H61B	0.9800
C43—C44	1.382 (2)	C61—H61C	0.9800
C44—C45	1.392 (3)		
			
C2—N1—C6	122.71 (13)	C41—C4—H4	108.00
C2—N3—C4	122.78 (12)	C15—C14—H14	108.00
C6—N1—H1	117.0 (14)	C16—C14—H14	108.00
C2—N1—H1	118.5 (14)	C44—C14—H14	108.00
C2—N3—H3	118.4 (13)	C14—C15—H15A	109.00
C4—N3—H3	117.6 (14)	C14—C15—H15B	109.00
N1—C2—N3	115.51 (13)	C14—C15—H15C	109.00
S2—C2—N3	123.78 (12)	H15A—C15—H15B	109.00
S2—C2—N1	120.71 (11)	H15A—C15—H15C	109.00
N3—C4—C41	110.05 (12)	H15B—C15—H15C	109.00
C5—C4—C41	113.87 (12)	C14—C16—H16A	109.00
N3—C4—C5	107.72 (11)	C14—C16—H16B	109.00
C4—C5—C6	117.84 (13)	C14—C16—H16C	109.00
C4—C5—C51	118.63 (12)	H16A—C16—H16B	110.00
C6—C5—C51	123.37 (13)	H16A—C16—H16C	109.00
N1—C6—C61	112.76 (13)	H16B—C16—H16C	109.00
C5—C6—C61	128.30 (14)	C41—C42—H42	120.00
N1—C6—C5	118.89 (13)	C43—C42—H42	120.00
C15—C14—C16	110.16 (17)	C42—C43—H43	119.00
C15—C14—C44	110.36 (16)	C44—C43—H43	119.00
C16—C14—C44	112.27 (16)	C44—C45—H45	119.00
C42—C41—C46	118.48 (16)	C46—C45—H45	120.00
C4—C41—C42	120.06 (14)	C41—C46—H46	120.00
C4—C41—C46	121.43 (14)	C45—C46—H46	120.00
C41—C42—C43	120.28 (16)	C51—C52—H52A	109.00
C42—C43—C44	121.41 (16)	C51—C52—H52B	109.00
C14—C44—C43	121.18 (16)	C51—C52—H52C	109.00
C14—C44—C45	120.86 (16)	H52A—C52—H52B	109.00
C43—C44—C45	117.95 (16)	H52A—C52—H52C	109.00
C44—C45—C46	121.05 (17)	H52B—C52—H52C	109.00
C41—C46—C45	120.78 (16)	C6—C61—H61A	109.00
O51—C51—C52	120.17 (13)	C6—C61—H61B	109.00
C5—C51—C52	117.29 (13)	C6—C61—H61C	109.00
O51—C51—C5	122.53 (13)	H61A—C61—H61B	109.00
N3—C4—H4	108.00	H61A—C61—H61C	109.00
C5—C4—H4	108.00	H61B—C61—H61C	109.00
			
C6—N1—C2—S2	163.66 (11)	C51—C5—C6—C61	4.0 (2)
C6—N1—C2—N3	−15.1 (2)	C4—C5—C51—O51	−164.54 (13)
C2—N1—C6—C5	21.1 (2)	C4—C5—C51—C52	16.63 (19)
C2—N1—C6—C61	−161.20 (13)	C6—C5—C51—O51	20.2 (2)
C4—N3—C2—S2	162.66 (11)	C6—C5—C51—C52	−158.59 (14)
C4—N3—C2—N1	−18.6 (2)	C15—C14—C44—C43	107.02 (19)
C2—N3—C4—C5	41.10 (18)	C15—C14—C44—C45	−71.9 (2)
C2—N3—C4—C41	−83.58 (17)	C16—C14—C44—C43	−129.67 (19)
N3—C4—C5—C6	−32.96 (17)	C16—C14—C44—C45	51.4 (2)
N3—C4—C5—C51	151.56 (12)	C4—C41—C42—C43	176.53 (15)
C41—C4—C5—C6	89.38 (16)	C46—C41—C42—C43	−1.7 (2)
C41—C4—C5—C51	−86.10 (15)	C4—C41—C46—C45	−176.17 (16)
N3—C4—C41—C42	−92.42 (16)	C42—C41—C46—C45	2.0 (3)
N3—C4—C41—C46	85.75 (17)	C41—C42—C43—C44	−0.5 (3)
C5—C4—C41—C42	146.53 (14)	C42—C43—C44—C14	−176.54 (16)
C5—C4—C41—C46	−35.3 (2)	C42—C43—C44—C45	2.4 (3)
C4—C5—C6—N1	5.98 (19)	C14—C44—C45—C46	176.88 (18)
C4—C5—C6—C61	−171.27 (13)	C43—C44—C45—C46	−2.1 (3)
C51—C5—C6—N1	−178.77 (13)	C44—C45—C46—C41	−0.2 (3)

### Hydrogen-bond geometry (Å, º)

**Table:**

*D*—H···*A*	*D*—H	H···*A*	*D*···*A*	*D*—H···*A*
N1—H1···O51^i^	0.83 (2)	2.12 (2)	2.9445 (17)	174 (2)
N3—H3···S2^ii^	0.840 (19)	2.56 (2)	3.3481 (13)	156.9 (18)
C61—H61*A*···O51	0.98	2.22	2.9148 (19)	127
C61—H61*B*···S2^iii^	0.98	2.86	3.7145 (16)	146

Symmetry codes: (i) *x*, −*y*+1, *z*−1/2; (ii) −*x*, −*y*, −*z*; (iii) −*x*, −*y*+1, −*z*.
